# Mixed Nodule Infection in *Sinorhizobium meliloti*–*Medicago sativa* Symbiosis Suggest the Presence of Cheating Behavior

**DOI:** 10.3389/fpls.2016.00835

**Published:** 2016-06-13

**Authors:** Alice Checcucci, Elisa Azzarello, Marco Bazzicalupo, Marco Galardini, Alessandra Lagomarsino, Stefano Mancuso, Lucia Marti, Maria C. Marzano, Stefano Mocali, Andrea Squartini, Marina Zanardo, Alessio Mengoni

**Affiliations:** ^1^Department of Biology, University of FlorenceFlorence, Italy; ^2^Department of Agri-Food Production and Environmental Science, University of FlorenceFlorence, Italy; ^3^European Molecular Biology Laboratory – European Bioinformatics Institute, Wellcome Trust Genome CampusCambridge, UK; ^4^Consiglio per la Ricerca in Agricoltura e l’Analisi dell’Economia Agraria, Centro di Ricerca per l’Agrobiologia e la PedologiaFlorence, Italy; ^5^Department of Agronomy, Food, Natural Resources, Animals and the Environment, University of PaduaPadova, Italy

**Keywords:** *Medicago sativa*, symbiotic nitrogen fixation, cheating, mixed nodules, competition, *Sinorhizobium meliloti*

## Abstract

In the symbiosis between rhizobia and legumes, host plants can form symbiotic root nodules with multiple rhizobial strains, potentially showing different symbiotic performances in nitrogen fixation. Here, we investigated the presence of mixed nodules, containing rhizobia with different degrees of mutualisms, and evaluate their relative fitness in the *Sinorhizobium meliloti*–*Medicago sativa* model symbiosis. We used three *S. meliloti* strains, the mutualist strains Rm1021 and BL225C and the non-mutualist AK83. We performed competition experiments involving both *in vitro* and *in vivo* symbiotic assays with *M. sativa* host plants. We show the occurrence of a high number (from 27 to 100%) of mixed nodules with no negative effect on both nitrogen fixation and plant growth. The estimation of the relative fitness as non-mutualist/mutualist ratios in single nodules shows that in some nodules the non-mutualist strain efficiently colonized root nodules along with the mutualist ones. In conclusion, we can support the hypothesis that in *S. meliloti*–*M. sativa* symbiosis mixed nodules are formed and allow non-mutualist or less-mutualist bacterial partners to be less or not sanctioned by the host plant, hence allowing a potential form of cheating behavior to be present in the nitrogen fixing symbiosis.

## Introduction

In the last decades, the interest on social interaction strategies of bacteria, including antagonism and cooperation has increased (Kiers et al., 2006). Nitrogen fixing symbioses between rhizobia and leguminous plants provide interesting models to study social dynamics of strains, which compete for entering in symbiosis with the same host plant (Denison and Kiers, 2004). Rhizobia interact with plant roots after the perception of flavonoid molecules released by the plant roots. In response to flavonoid, lipo-chito-oligosaccharide molecules (Nod Factors) are produced by rhizobia and trigger a molecular pathway on plant cells. This mechanism ultimately leads to rhizobial entry into plant root tissues, intracellular colonization, formation of the root nodule structure, and differentiation of intracellular rhizobia in bacteroids. Then, bacteroids will express the nitrogenase genes responsible for the fixation of atmospheric di-nitrogen to ammonium, thus providing a selective advantage to plants growing in nitrogen-depleted soils.

The plant–rhizobia relationship is not exclusive: multiple rhizobial strains colonize the same individual plant (Kiers et al., 2006), and large genetic differences in strains isolated from the very same root apparatus have been reported (Carelli et al., 2000). Then, in theory, plants can enter into symbiosis with rhizobial strains having different symbiotic performances (e.g., capacity to fix di-nitrogen, to colonize root nodules, etc.). To control the efficiency of the symbiotic partnership and then avoid overcolonization by strains fixing low amounts of nitrogen, plants have evolved mechanisms which favor nodules colonized by rhizobia supplying the host with a higher amount of nitrogen (Kiers et al., 2003). In fact, the plant seems to monitor bacterial performances and states sanctions on nitrogen fixation defective strains, penalizing them in the colonization (Kiers et al., 2003). However, in nature, strains belonging to the same species but with different levels of symbiotic performances are present. Such differences have also a practical importance in agronomy, since they are the basis for the selection of ‘élite’ inoculant strains to be applied for legume yield improvement (Dwivedi et al., 2015). Deciphering the competition patterns among natural strains and the selection by host plant would be of great importance for inoculant strains development.

An additional level of competition occurs at single nodule level. Several studies have reported the occurrence of a bacterial community composed by symbiotic rhizobia and by other (apparently non-symbiotic) strains in soybean, common bean, cowpea, and clover (Moawad and Schmidt, 1987; Mårtensson et al., 1989; Sessitsch et al., 1997; Denison, 2000). The colonization of the same nodule by different symbiotic strains may allow to establish a sort of cooperation between symbiotic rhizobia to overcome plant sanctions in the case of non-mutualist (e.g., which do not fix atmospheric nitrogen) or less-mutualist rhizobial partners (e.g., which have a limited nitrogenase functionality), but also may pave the way to ‘free riders’ or cheaters, which can be masked against sanctions directed toward the whole nodule, as shown on Bradyrhizobia (Denison, 2000).

The partnership between the rhizobium *Sinorhizobium meliloti* and legumes of genus *Medicago* (alfalfa and relatives) is one of the most investigated model systems of symbiotic nitrogen fixation. In this interaction, rhizobia are terminally differentiated into bacteroids inside root nodules (indeterminate nodules) and lose the ability to reproduce (Sprent, 2001; Gibson et al., 2008). However, indeterminate nodules also contain significant numbers of undifferentiated rhizobia, which can reproduce within the nodule tissues and are released after nodule senescence (Denison and Kiers, 2004). Therefore, a mixed rhizobial population inside the same nodule can be present, with both terminally differentiated and undifferentiated cells. The mixed (differentiated and undifferentiated) rhizobial population within the nodule may pose a question about the possibility of effective sanctions (Oono et al., 2009). Indeed, split-root experiments performed on *Sinorhizobium–Medicago* nodules did not support the hypothesis of sanctions over single nodules but indicate that a plant choice, occurring at some stage during infection, could be present (Gubry-Rangin et al., 2010). In this perspective, if single nodules are not sanctioned, we can hypothesize that the presence of multiple rhizobial strains in the same nodule with different level of mutualism (i.e. with different degrees of nitrogen fixation), would be more favored in indeterminate nodules (as those of *S. meliloti – Medicago*) than in determinate ones. Consequently, we can expect free-riding or cheating to be present in *S. meliloti*–*Medicago* symbiosis.

This work therefore aims at in providing experimental evidence on the presence of mixed single nodules and on the possibility of cheating/free riding to occur by non-mutualist strains in the *S. meliloti*–*Medicago* symbiosis. To fulfill this aim we evaluated:

(i) the presence of mixed nodules, which contain both the mutualist and the non-mutualist strain;

(ii) the differences in strains competitiveness in terms of nitrogen fixers nodules formed;

(iii) the fitness of the different strains in single and mixed nodules, in terms of number of bacterial cells present in nodules.

Two natural strains (plus the mutualist laboratory strain *S. meliloti* 1021) were selected for this study, a mutualist (BL225C) and a non-mutualist strain (AK83; Biondi et al., 2009) In particular, *S. meliloti* AK83 strain is able to form symbiotic nodules on alfalfa (*M. sativa*) but nitrogen fixation does not take place, either for the lack of a large gene set on the symbiotic megaplasmid pSymA (Galardini et al., 2011) and/or for the presence of an accessory plasmid which contains functions related to nitrogen fixation blocking (Crook et al., 2012; Price et al., 2015) Based on these premises, *S. meliloti* AK83 represents a good test strain for a free-riding/cheating behavior inside mixed root nodules.

## Materials and Methods

### Bacterial Strains, Plasmids, and Growth Conditions

The strains and plasmids used in this work are listed in **Table 1**. *S. meliloti* strains were cultured on solid or liquid tryptone yeast (TY) medium with 0.2 g/l CaCO_3_, while *Escherichia coli* strains were grown in Luria Bertani (LB) medium, supplemented with antibiotics when necessary.

**Table 1:** Strains and plasmids used.

Strains (or plasmids)	Species	Description	References
1021	*Sinorhizobium meliloti*	Str^r^ derivative from strain 2011. Mutualist	Meade et al., 1982
AK83	*S. meliloti*	Lacks part of the microaerophilic gene set on pSymA-homolog megaplasmid. Non-mutualist	Galardini et al., 2011
BL225C	*S. meliloti*	Mutualist	Galardini et al., 2011
BM102	*S. meliloti*	Rif^r^ derivative of *S. meliloti* AK83. Non-mutualist	This work
BM267	*S. meliloti*	Rif^r^ derivative of *S. meliloti* BL225C. Mutualist	This work
BM685	*S. meliloti*	*S. meliloti* AK83 pBHR mRFP (Rif^r^ Tet^r^). Non-mutualist	This work
BM687	*S. meliloti*	*S. meliloti* 1021 pBHR mRFP (Str^r^ Tet^r^). Mutualist	This work
BM325	*S. meliloti*	*S. meliloti* AK83 pHC60 (Rif^r^ Tet^r^). Non-mutualist	This work
BM257	*S. meliloti*	*S. meliloti* 1021 pHC60 (Str^r^ Tet^r^). Mutualist	This work
BM286	*S. meliloti*	*S. meliloti* BL225C pHC60 (Rif^r^ Tet^r^). Mutualist	This work
S17-1 λpir	*Escherichia coli*	Tp^r^, Sm^r^, *recA*, thi, *hsdR*^-^M^+^, RP4::2-Tc::Mu::Km::Tn7, λpir lysogen	Simon et al., 1983
BM679	*E. coli*	*E. coli* S17-1 λpir pBHR mRFP (Tet^r^)	This work
BM266	*E. coli*	*E. coli* S17-1 λpir pHC60 (Tet^r^)	This work
pBHR mRFP		Constitutive expression of RFP, Tet^r^	Smit et al., 2005
pHC60		Constitutive expression of GFP, Tet^r^	Cheng and Walker, 1998

### Preparation of Fluorescently Tagged Strains and CLSM Imaging

Strains were tagged with green fluorescent protein (GFP) or red fluorescent protein (RFP). Plasmids pHC60 (harboring a constitutively expressed GFP; Cheng and Walker, 1998) and pBHR mRFP (harboring a constitutively expressed RFP; Smit et al., 2005) were used for transformation of *E. coli* S17-1 cells. Transformants were selected by resistance to Tet (10 μg/ml). Positive clones were used for biparental conjugation to a rifampicin-resistant derivative of *S. meliloti* AK83 and to *S. meliloti* 1021 (resistant to streptomycin). Conjugal transfer was performed as previously described (Pini et al., 2013b). Confocal imaging was performed on fresh cut nodules 4 weeks after inoculums, using an upright Leica Laser Scanning Confocal Microscope SP5 (Leica Microsystems, Germany) equipped with a 63 × oil immersion objective. Imaging of GFP and RFP were performed using 488-nm excitation of an argon laser line for GFP and 543-nm He/Ne for RFP.

### Nodulation and Nitrogen Fixation Assays

Seedlings of *Medicago sativa* (cv. Pomposa) were sterilized in HgCl_2_, repeatedly washed, and germinated in sterile plastic Petri dishes for 72 h in the dark and 48 h in the light at room temperature. For *in vitro* assays, seedlings were transferred in Petri dishes containing Buffered Nod Medium (Biondi et al., 2009) and 16 g/l of type A agar (Sigma–Aldrich). Plantlets were grown for an additional 3–5 days before inoculation with *S. meliloti* strains. For nodulation assays, strains were grown in liquid TY medium at 30°C for 48 h, then washed three times in 0.9% NaCl solution and resuspended to an OD_600__nm_ of 1.0. 1 × 10^7^ cells (in single or a 1:1 ratio for competition experiments) were used (corresponding to 4 × 10^4^ cells/cm^2^). Cells were directly spread over the seedling root. Plates were pierced to let the plant grow outside, and transferred in a near-vertical position to a growth chamber maintained at 26°C with a 16-h photoperiod (100 microeinstein/m^2^ s).

Microcosm-scale nodulation assays were performed in plastic pots containing approximately 400 g of native (unsterilized) sandy-clay soil from the university campus garden. Each pot was sown with four surface-sterilized seeds and after germination rhizobial strains (grown and washed as described for the *in vitro* assays) were inoculated into the pot to a final concentration of ca. 2 × 10^4^ cells/g of soil, similar to the rhizobial estimates in agricultural soil. Plants were then maintained at 26°C with a 16-h photoperiod (100 microeinstein/m^2^ s).

Nitrogen fixation rates were measured by the acetylene-reduction assay and expressed in nanomoles of produced ethylene per hour, per plant, as previously described in Muresu et al. (2008). All measures were taken 4 weeks after rhizobial inoculums, as commonly reported for *S. meliloti–Medicago* symbiotic tests (see for instance Yendrek et al., 2010; Pini et al., 2013a,b). Statistical analysis of data was performed with nonparametric Kruskal–Wallis test by Analyse-it software ver. 2.0 (Analyse-it Software, Ltd). Additional tests and figures were generated through Python scripts using the Numpy, Scipy (Van der Walt et al., 2011), Pandas (McKinney, 2010), Matplotlib (Hunter, 2007), and Seaborn (Waskom et al., 2015) libraries (**Supplementary Material S1**).

### Estimation of Bacterial Loads in Nodules

Bacterial loads have been estimated by both a cultivation and a molecular (DNA-based) method. For cultivation, single (at least 10 days old, size >1 mm) nodules were excised from plants, surface sterilized with 0.1% NaHClO for 30″, washed three times in sterile distilled water, crushed and re-suspended in 100 μl of 0.9% NaCl sterile solution. Aliquots of serial dilutions and the third wash water (as control) were then plated on TY plates and incubated at 30°C for 48 h. The numbers of CFU were used for titration of viable and culturable cells. For the molecular method, bacterial DNA was extracted from surface-sterilized single nodules (as mentioned above) by using a fast protocol for plant DNA extraction (Edwards et al., 1991). Real-time PCR was performed by applying a previously published protocol (Trabelsi et al., 2009), based on the *S. meliloti* primer pair for core genome rpoE1 gene and on strain-specific primer pairs identified after comparative genome analysis (Galardini et al., 2011). In particular, for 1021, a strain-specific primer on Smc01419 gene (fw-5′-CGAGGAAGAGGTCCTGGAAT-3′, rv-5′-GACGCAGTCCTGCAACAGAT-3′) was designed, for AK83 strain on gene SINME_RS34005 (old locus tag Sinme_6912) (fw-5′-GATTTTCCGCGACTCTGAAG-3′, rv-5′-AGTCCGGTGTCAGATTCAGG-3′), and for BL225C on gene SinmeB 5863 (fw-5′GAAGCAGATGCTATCGGCAC-3′, rv-5′-TλACAGCACCACAGGCGAC-3′). All mentioned genes are single copy genes. Real-time (qPCR) was performed in an QuantStudio^TM^ 7 apparatus (Applied Biosystems) programmed with the following temperature profile: 2 min 94°C, followed by 40 cycles composed by 15 s 94°C, 15 s 63°C, 30 s 72°C. Fluorescence data acquisition was done during the extension step at 72°C. A final melting curve was performed to check for product specificity. Reactions were performed in 10 μl final volume containing 5 μl of SYBR Green mix (Thermo Fisher Scientific Maxima SYBR Green/ROX qPCR Master Mix (2×), 0.5 μl ROX solution (included in the kit) and 10 pmol of primers. All reactions were done in triplicate. Standard curves with serial dilutions (1 ng–0.1 pg) of purified DNA from 1021, AK83, and BL225C were included. Data were analyzed with the QuantStudio^TM^ 7 Flex System software (Applied Biosystems) using fast 96-Well Block (0.1 ml). PCR efficiency was calculated as in Bar et al. (2003). Bacterial cell number was estimated as genome copies (considering one genome copy per cell) present in a given DNA amount (genome sizes for strains are those derived from genome sequencing data (PRJNA42477 for BL225C, PRJNA41993 for AK83, PRJNA57603 for 1021).

Competition index (CI) was evaluated as log of the ratio of CFU or qPCR estimates between the two strains. by using a conventional metrics (Ellermeier et al., 2006; Crook et al., 2012) In particular, CI = log (titre of strain A recovered/titre of strain B recovered)/(titre of strain A inoculated/titre of strain B inoculated).

## Results

### *S. meliloti* – *M. sativa* Symbiosis Allows a Large Number of Mixed Nodules

The common model of symbiotic interaction wrongly dictates the monoclonality of symbiotic rhizobia in root nodules, i.e. one nodule is colonized by one rhizobium strain only (Denison, 2000). Here, we provide evidence that both *in vitro* and in soil conditions, root nodules, formed after inoculating with a mixture of two rhizobial strains, contain both strains and that such nodules (when they include both mutualist and non-mutualist strains) fix nitrogen at rates similar to root nodules formed by the sole mutualist strain.

**FIGURE 1:**
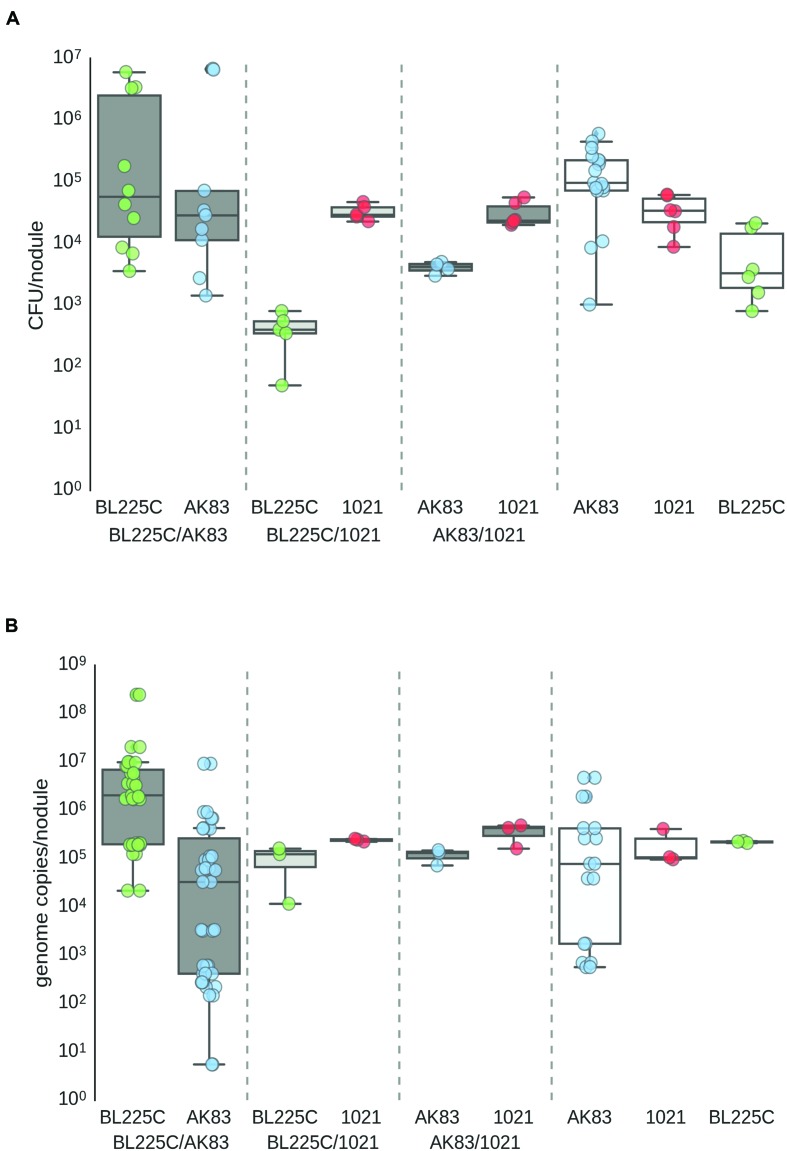
**Titres of *Sinorhizobium meliloti* on nodules (A) Viable titres estimates, (B) qPCR estimates.** Viable cells/nodules in mixed nodules from competition experiments and single strain inocula (controls). Box-and-Whisker plots are from 3 to 20 single nodules, each collected from a different plant. Data are from plate experiment.

First, we provided a quantitative estimation of the frequency of nodules with mixed infection in *M. sativa* plants under *in vitro* conditions. Single nodules were crushed, plated on TY plates and subjected to DNA extraction. Colonies grown on plates and extracted DNA from nodules were then analyzed by PCR with strain-specific primers (see MATERIALS AND METHODS). Results showed the presence of mixed nodules in all assays (20 plants with 3 nodules/plant for each competition). Titres estimated by plating and qPCR ranged from 10^1^ to 10^6^ cells/nodule (**Figures 1A,B**). In soil nodulation assays, performed by inoculating garden soil with the rhizobial culture, confirmed the *in vitro* results, suggesting that in nature *S. meliloti* may indeed form mixed nodules with *M. sativa* (**Supplemental Figure S1**).

**FIGURE 2:**
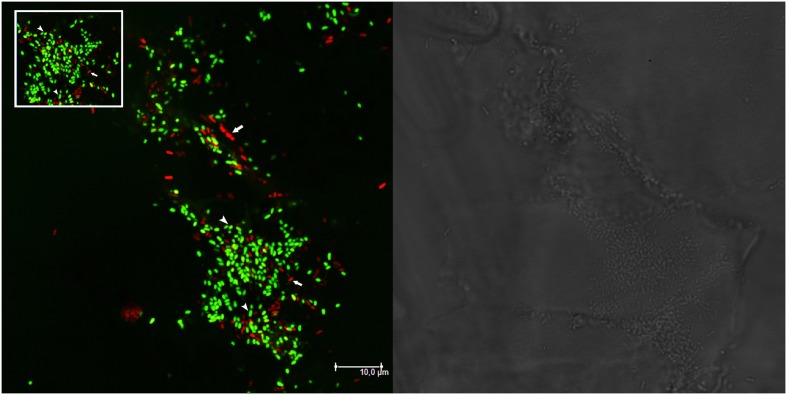
**Nodules may be colonized by different strains.** Confocal laser scanning microscopy (CLSM) image of a portion of root nodule containing both BM687 (*S. meliloti* 1021 pBHR mRFP, in red) and BM286 (*S. meliloti* BL225C pHC60, in green) strains. Data from plate experiment. The bright field layer and the CLSM layer are shown. Arrows in the CLSM panel indicate both strains (in red and in green) colonizing the nodule. The inset highlights the central part of the CLSM image with BM687 and BM286 strains.

**FIGURE 3:**
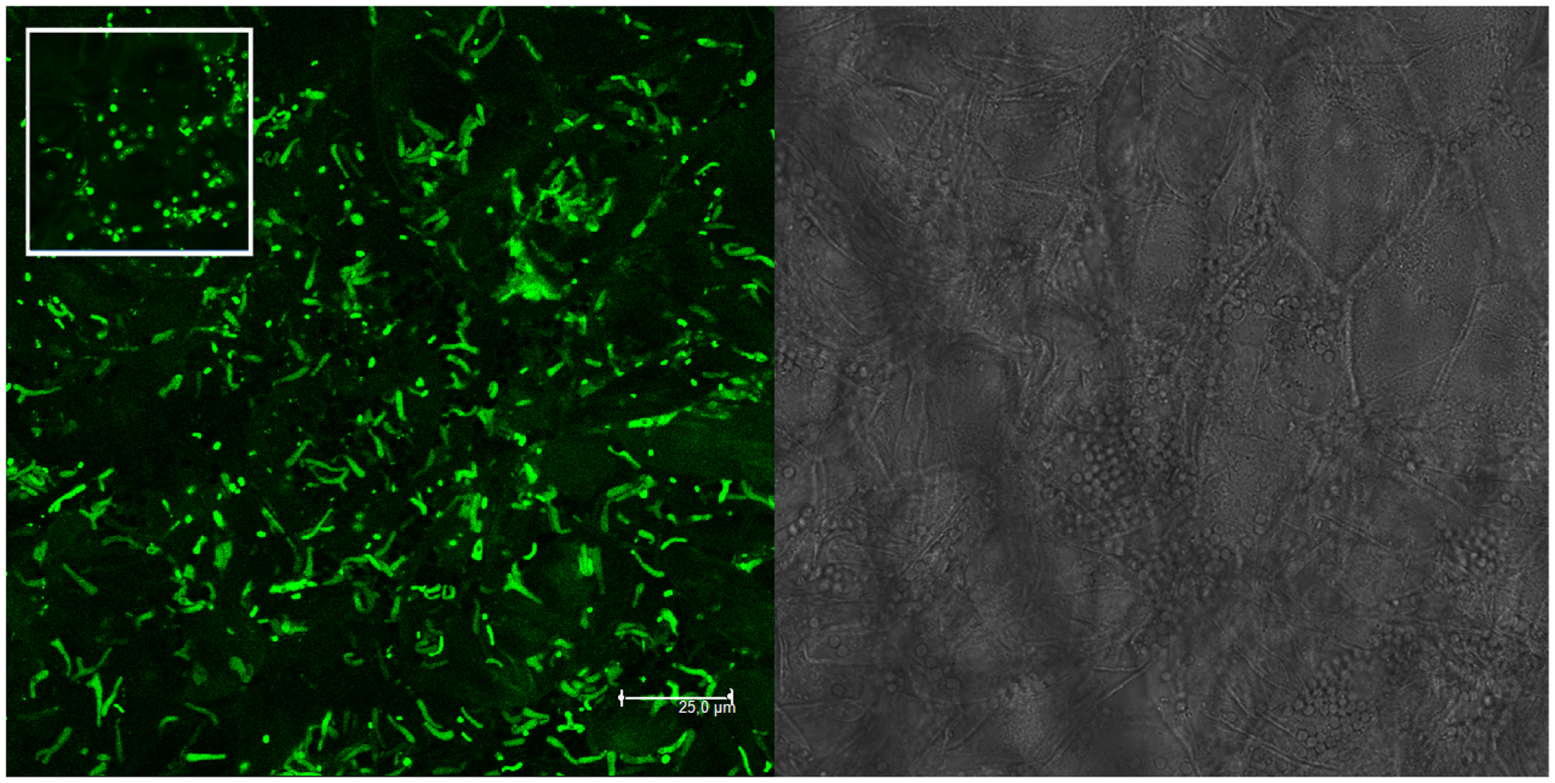
**Non-mutualist strain AK83 colonizes root nodules and undergo bacteroid differentiation.** CLSM image of BM325 strain (*S. meliloti* AK83 pHC60) inside root nodules of *M. sativa* plants co-infected *in vitro* with strains BM325 and BM286. Y-shaped bacteroid-like cells of BM325 strain are abundant. The bright field layer and the CLSM layer are shown. The inset highlights a portion of the image were non-differentiated cells are present (as round-shaped cells).

Molecular and plating data about the presence of mixed nodules were confirmed also by microscopy analysis on single *M. sativa* nodules colonized by GFP- and RFP-tagged derivatives of the strains (**Table 1**). Nodules with both strains present (AK83 and BL225C, as well as AK83 and 1021 or BL225C and 1021) were detected (**Figure 2**). Interestingly, both rhizobial strains were in non-differentiated form (normal bacterial-shape) in the meristematic part of the nodule and as intracellular bacteroid-shaped cells which can fix nitrogen (morphologically, thickened and long, Y-shaped cells surrounded by a membrane), also for the non-mutualist AK83 strain (**Figure 3**).

**FIGURE 4:**
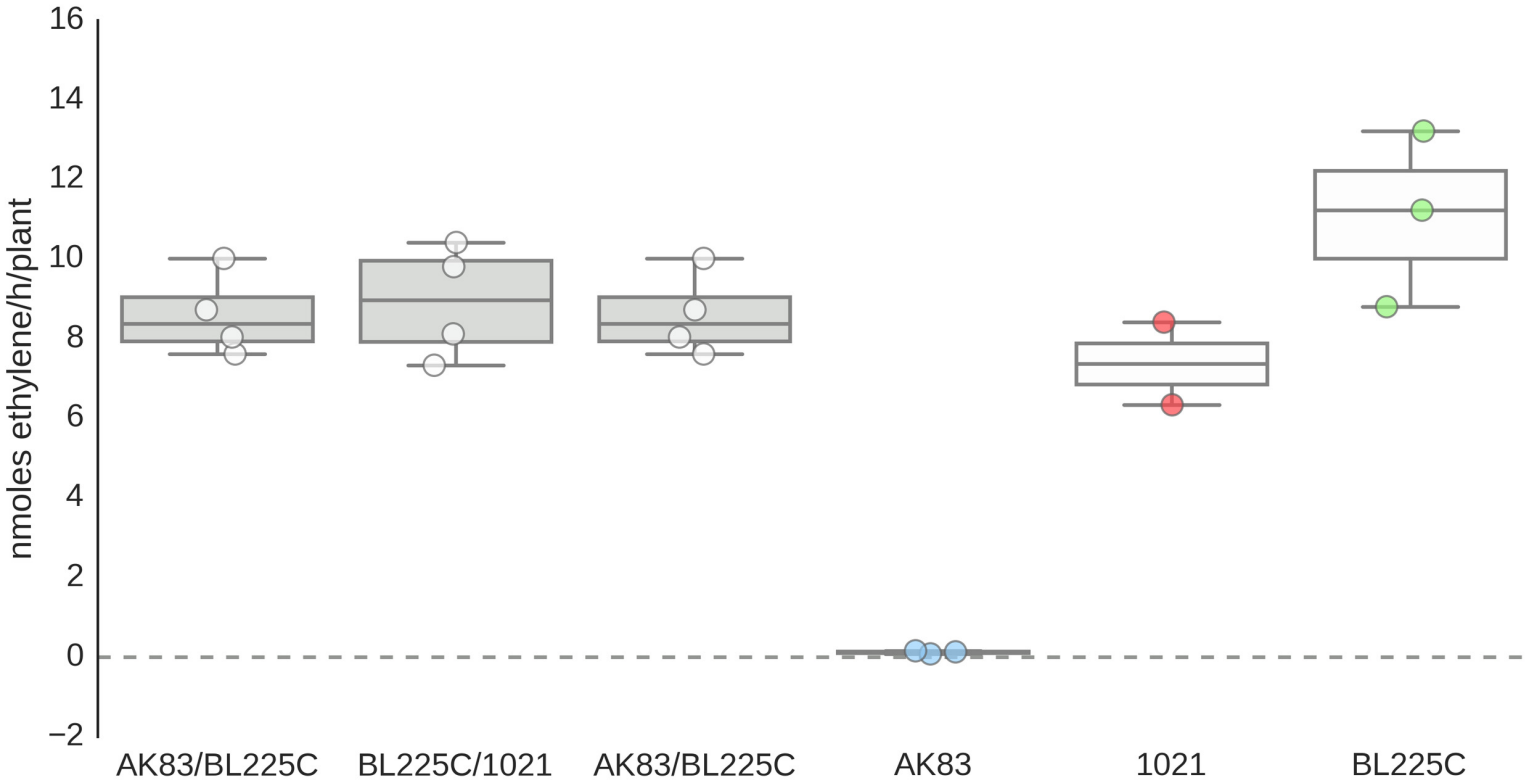
**Nitrogen fixation efficiency of nodules.** Reduction assay results expressed in terms of nmoles ethylene/h/plant. Box-and-Whisker plots are from 3 to 4 nodules each from different plant.

Finally, when the physiological efficiency of nodules was considered in terms of fixed nitrogen rates (acetylene-reduction assay), it emerged that the nitrogen fixation rate is not significantly reduced by the presence of the non-mutualist AK83 along with a mutualist strain (BL225C or 1021) (or, if it has a very faint effect, it is not statistically significant after Kruskal–Wallis test; **Figure 4**) in agreement with nodulation score and nodulation index which indicate that the presence of mixed infection in nodules, seems not to have a strong influence on plant growth (**Supplemental Figure S2**). From these data, we can support the hypothesis that the non-mutualist phenotype in terms of nitrogen fixation can be masked in mixed nodules by the nitrogen fixation rate of the mutualist partner, thus avoiding plant sanctions toward the formation of mixed nodules. In particular, we can hypothesize that the good nitrogen fixation rates of mixed nodules can be due to an over-dominance of bacteroids of the mutualist strain with respect to the non-mutualist. Consequently, the number of rhizobial cells contained in single nodules was estimated.

### The Non-mutualist Strain Eeficiently Colonizes Root Nodules

**FIGURE 5:**
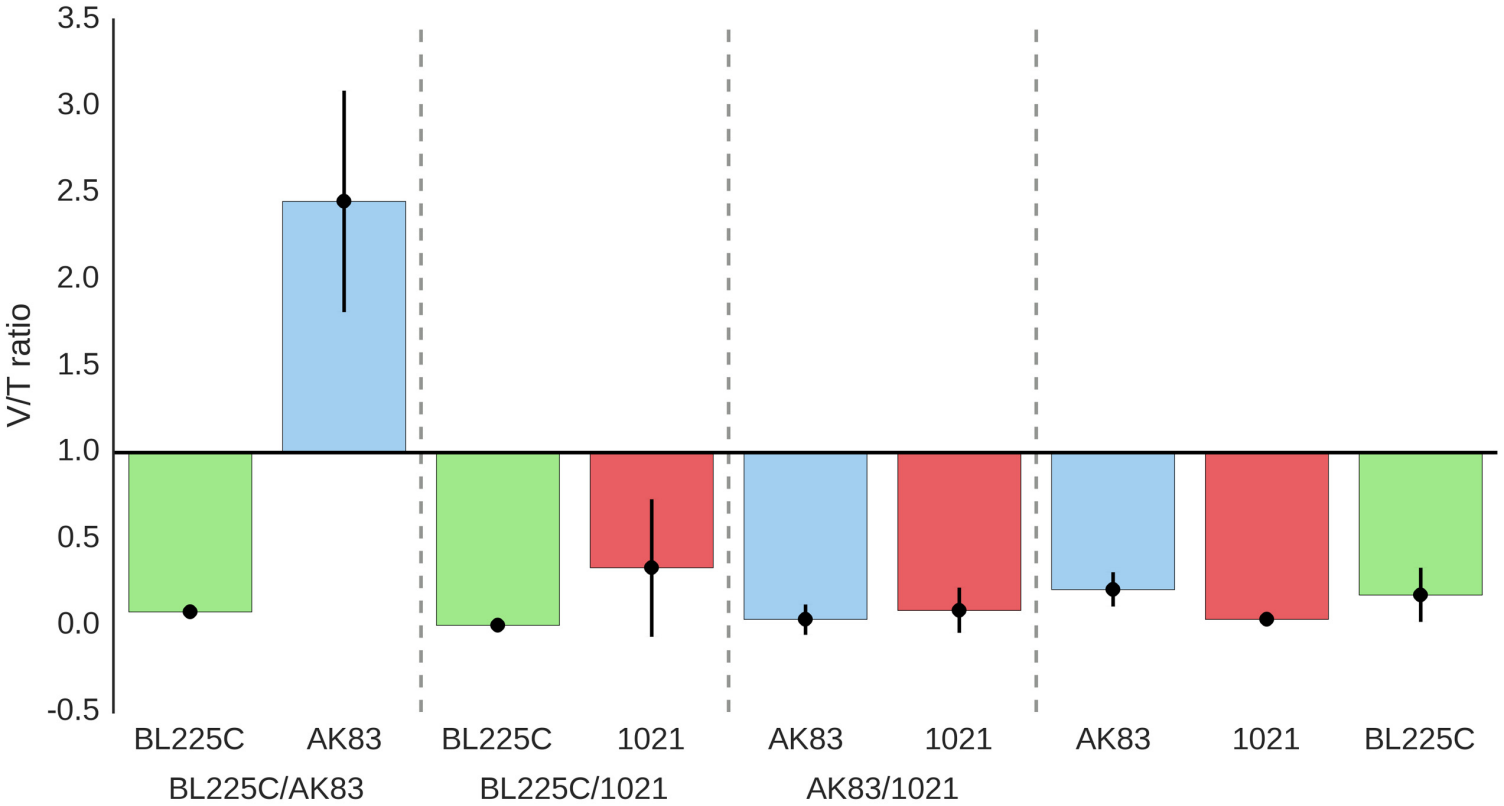
**Average values of viable/qPCR ratio as a proxy of estimates of bacteroid differentiation.** The ratio between viable and qPCR estimates for the same nodules are reported. The nodules tested (from 3 to 20) are from different plants.

The amount of cells present in each nodule was considered as a proxy for fitness, as widely reported (see for instance (Kiers et al., 2003; Ratcliff et al., 2011). Both viable cells (after plating on TY plates) and total *S. meliloti* cells (estimated by qPCR) were counted on each nodule from single and mixed inocula. In most of the experiments, qPCR titres were higher (1–2 log) than viable titres (ratio < 1; **Figures 1A,B**). Although this difference might be due to technical reasons (difference in sensitivity between plating and qPCR), we cannot exclude that the consistently higher values of qPCR estimates may be due to the presence of the terminally differentiated bacteroids (which cannot be cultivated and contain multiple genome copies; Mergaert et al., 2006). Under this hypothesis, the ratio between viable and qPCR titres estimates was used to provide a possible proxy of the amount of strain differentiation within nodules (**Figure 5**). In the competition experiments between the mutualist BL225C and the non-mutualist AK83, viable/qPCR ratio was >1 for AK83, while approximated <1 for BL225C. These data let to hypothesize that the plant may favor the differentiation of the mutualist strain (BL225C). Such difference between the two competitors was not observed in the competition experiment between the two mutualist strains (as BL225C vs. 1021) as well as between 1021 and AK83 (values were below 1 for all strains). A similar higher colonization level of the mutualist strains (BL225C and 1021) with respect to strain AK83 was also observed in the soil experiment (**Supplemental Figure S1**). These results could be in agreement with a model of control over bacteroid differentiation operated by the plant (e.g., through NCR peptides), which favors the differentiation of mutualist strains, then maximizing the fixed nitrogen during symbiosis (Van de Velde et al., 2010; Price et al., 2015). However, it is still obscure whether some genetic determinant in the mutualist may positively affect the overall differentiation in the competition, similarly to the host range restriction peptidase (*hrrP*) present in the non-mutualist AK83 (Crook et al., 2012; Price et al., 2015).

However, we cannot exclude that estimates of bacteroid differentiation obtained with other methods, e.g., based on flow cytometry (Ratcliff et al., 2011) and evaluation of rhizobial cells in nodules at different stages could clarify the biological interpretation of the observed variability in qPCR estimates.

**FIGURE 6:**
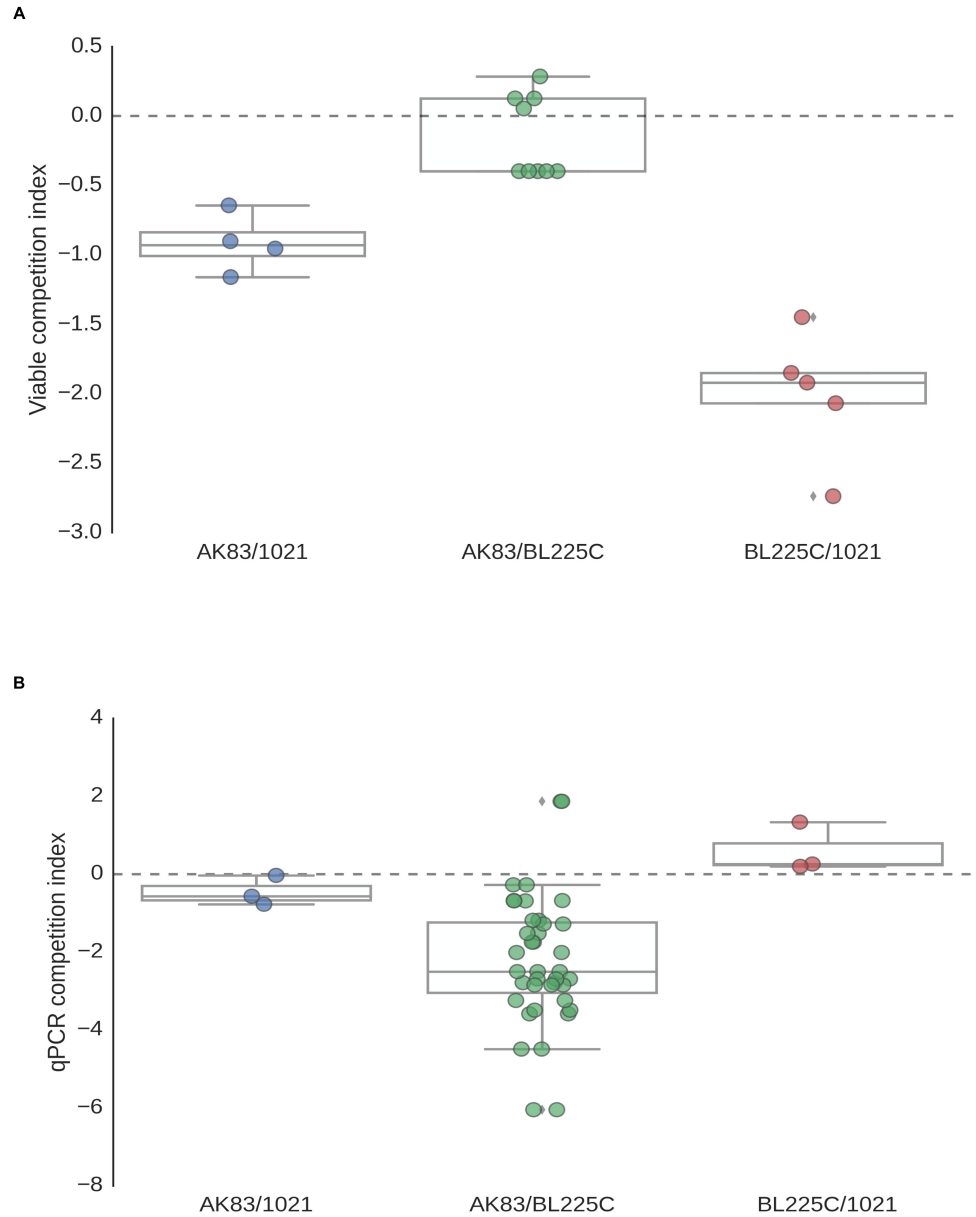
**Competition index in mixed nodules: viable (A) and qPCR (B) estimates of competition index for mixed nodules (from 3 to 20) coming from different plants.** Log titres ratios are reported in ordinates.

Competition indices (CI; **Figure 6**) showed that the non-mutualist strain AK83 was present, in many cases, at lower titres than the mutualist ones (BL225C and 1021, ratio <1). Then, in general, a lower fitness in competition of the non-mutualist with respect to the mutualist strain was detected, particularly in qPCR estimates (**Figure 6B**). However, nodules in which the non-mutualist colonized at titres higher than the mutualist strain were present, especially in the viable estimates of AK83 vs. BL225C competition (**Figure 6A**).

This pattern of competition was also confirmed in an *in vivo* test on garden soil, with the mutualist strain (1021 and BL225C) being dominant in nodules with respect to the non-mutualist one (AK83) (Supplemental Table S1).

## Discussion

In this work we have, for the first time, generated evidences of widespread presence of mixed infection in nodules which contain cheater strains in the *Sinorhizobium–Medicago* symbiosis.

From the analysis of the colonization patterns, we found that nodules may host more than one strain. In several cases the non-mutualist strain was present with low number of cells inside the mixed nodules, but nodules in which it was present at high titres (similar or even higher than those of the mutualist strain) were also found. Plants nodulated by both mutualist and non-mutualist did not show an appreciable decrease in plant growth, nor root nodules showed a significantly large decrease in nitrogen fixation rates compared to plants nodulated by mutualist strains. These results support the hypothesis that, in nodules with mixed infection, the nitrogen fixation phenotype of the mutualist strains (and consequently the effect on plant growth and, potentially, on plant fitness) is prevalent at the whole plant level. Consequently, the non-mutualist phenotype of some strains (as AK83) can be masked (against plant sanctions) by the fixed nitrogen provided by the mutualist strains. Then the non-mutualist strains can be protected against sanctions to ineffective (non-nitrogen fixing) nodules by the activity of the mutualist ones. After this consideration, we can support the hypothesis that a fraction of the rhizobial nodulating population could indeed be a cheater of the mutualist one (Denison, 2000; Denison and Kiers, 2004) or anyway may thrive, as free-rider, at the expenses of the mutualist strains. However, we cannot exclude that despite both viable and qPCR titres of nodule colonization indicate a dominance of the mutualist strains, a plant differential control over bacteroid formation could be present. In fact, different strains of *S. meliloti* (including AK83) seem to vary in their response to NCR peptides (Crook et al., 2012; Price et al., 2015), and consequently in the number of cells which do not terminally differentiate. In our experiments AK83 is forming bacteroid-shaped cells in *M. sativa* plants, even in mixed infection. This evidence indicates that the plant variety we used is producing NCR peptides able to differentiate AK83, then possibly limiting its infection as cheater. We can hypothesize that in other host plant species or other *M. sativa* varieties a different behavior of AK83 could be present, since AK83 strain harbors a *hrrP* homolog gene on pSINME01 plasmid (Price et al., 2015), which can selectively degrade some NCR peptides.

In general, the picture coming from the presented data is that the non-mutualist strains can have some space to thrive and multiply within the nodules of the root apparatus, without apparently reducing plant growth. The ability to multiply in the nodules (even if with reduced titres with respect to the mutualist strains) may allow to support the presence of a non-mutualist fraction in the rhizobia soil population (Nowak and Sigmund, 2002; Kiers et al., 2003). However, the consequences of this supposed cheating on plant fitness cannot be fully answered. In fact, although a “fitness” alignment has been reported between the rhizobia and the host plant (Friesen, 2012) and nodulation parameters have been shown to be correlated with plant biomass (Friesen, 2012), no measures of direct fitness (number of seeds, number of rhizobial cells released) were reported. Then, relevance of the presence of nodules with mixed infection containing non-mutualist strains on plant fitness has to be clarified. In fact, similar plant growth or nitrogen fixation rates could not imply directly similar number of seeds/offspring. Moreover, we cannot exclude that during nodule development the ratio of mutualist vs. non-mutualist strains may vary in relation to the differentiated and meristematic zone areas.

The impact of cheating on rhizobial evolution is hard to evaluate. Since rhizobial symbiosis is widespread in different bacterial taxa, even poorly related at the taxonomic level (cfr. the existence of alpha and beta-rhizobia), it is clear that symbiosis is conferring a fitness advantage for plant-associated bacteria (Remigi et al., 2014, 2016). However, the presence of other non-symbiotic ecological niches of rhizobia, as soil and plant endosphere, as well as the presence of genetic determinants modulating the competitiveness in different host plant varieties (Crook et al., 2012; Price et al., 2015) is puzzling in regard to the ecological and evolutionary relevance of cheating, with respect to the overall fitness of cheating strains. A strain behaving as cheater with one host plant genotype (variety, species, etc.) may be a good mutualist with a different plant genotype or having high fitness in a different environmental context (e.g., soil or rhizosphere). Moreover, the allowed presence of a supposed cheater might be based on other hitherto undetected phenotypes, different from nitrogen fixation, that could be beneficial for the plant. Indeed, it should be also kept in mind that, besides rhizobial cheaters, a high variety of endophytes of different taxonomy are regularly detected as co-occupants of nodules in many spontaneous legumes (Muresu et al., 2008; Zgadzaj et al., 2015). These endophytes may enter the nodule via Nod signaling from the true symbiotic strains (Zgadzaj et al., 2015) and may allow a fraction of non-symbiotic rhizobia to colonize root nodules formed by mutualistic rhizobia. However, the general role of root nodule non-symbiotic endophytes in affecting (positively or negatively) symbiotic nodule trophism is still unclear. Experiments in field conditions, evaluating both the nodulating and the non-nodulating fractions of the rhizobial population in competition experiments may allow to solve some of these questions.

## Author Contributions

AC performed competition experiments, *in vitro* symbiosis test produced recombinant strains and drafted the manuscript. EA, LM, SM performed CLSM experiments. AL and SM did phenotype testing on nodulated plants. AS and MZ performed nitrogen-fixation assays on nodules. MM settled the experimental protocol for competition experiments. MG performed the pangenome analysis of strains and prepared the figures. MB contributed in conceiving the idea. AM contributed in conceiving the idea and drafted the manuscript. All authors have read and approved the manuscript.

## Conflict of Interest Statement

The authors declare that the research was conducted in the absence of any commercial or financial relationships that could be construed as a potential conflict of interest.

## Supplementary material

FIGURE S1 **Number of copies of the genome/nodule of the qPCR estimates in the in vivo experiment.** The plants were inoculated with the wild type strains. Measurements were performed on three nodules for each mixed inoculum.

FIGURE S2 **Effect of strain competition on host plants. (A)** Nodulation index. Percentage of nodulated plants for single and mixed strains combinations. Values are means (śstandard deviation) of three independent experiments, each involving at least 20 plants. **(B)** Nodulation score. Mean number of nodules/plant. Values indicate means ś standard deviation of number of three independent experiments, each involving at least 20 plants. The only significant pairwise contrast is that AK83 vs. BL225C-AK83 (*P* < 0.05).

SUPPLEMENTAL MATERIAL S1 **Input data and code used to generate the figures**.
