# Supplementary figures and images for: Mixed Nodule Infection in Sinorhizobium meliloti–Medicago sativa Symbiosis Suggest the Presence of Cheating Behavior
Source: Front Plant Sci. 2016 Jun 13;7:835. doi: 10.3389/fpls.2016.00835 (PMC4904023; doi:10.3389/fpls.2016.00835)

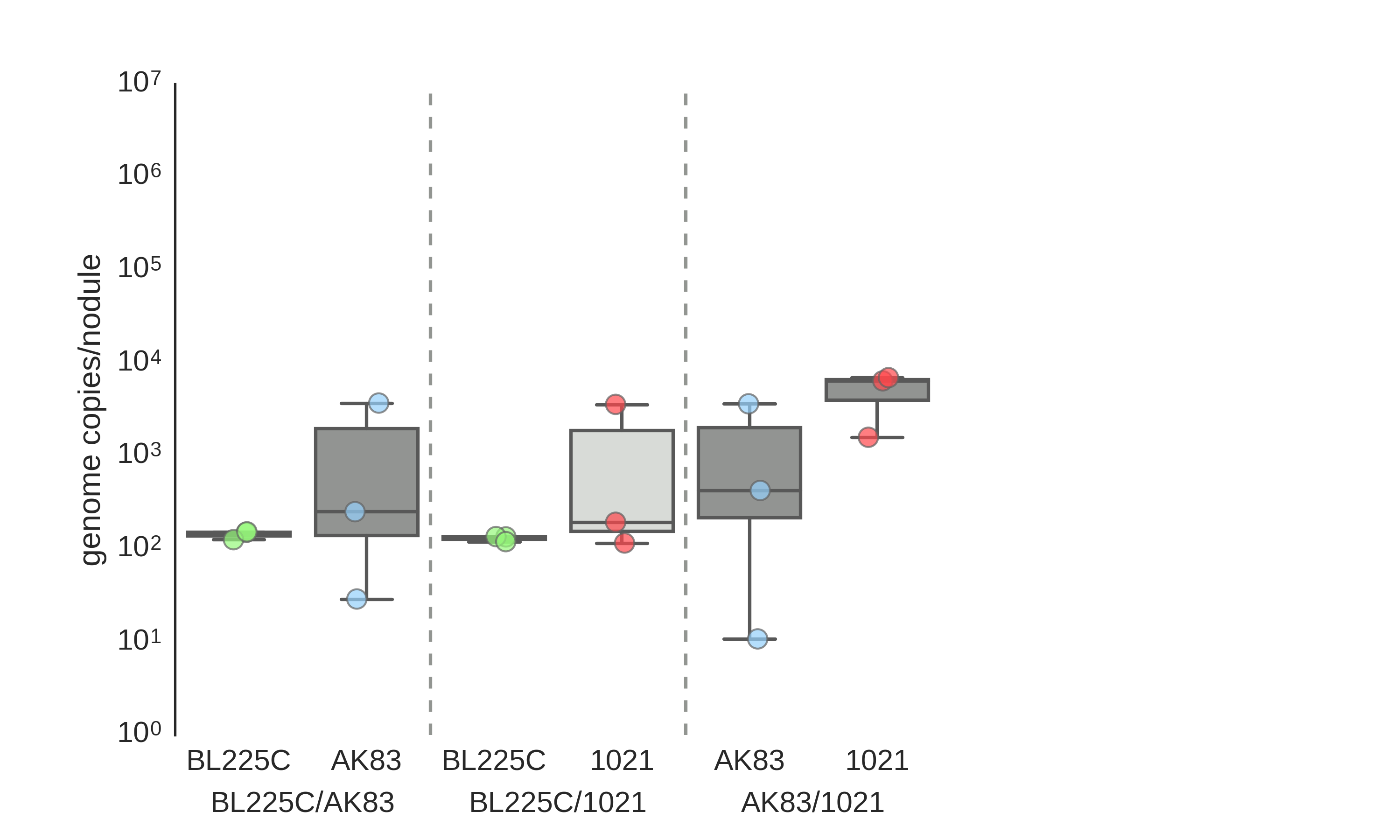

Supplement: FIGURE S1 — Number of copies of the genome/nodule of the qPCR estimates in the in vivo experiment. The plants were inoculated with the wild type strains. Measurements were performed on three nodules for each mixed inoculum. [file Image_1.TIF]

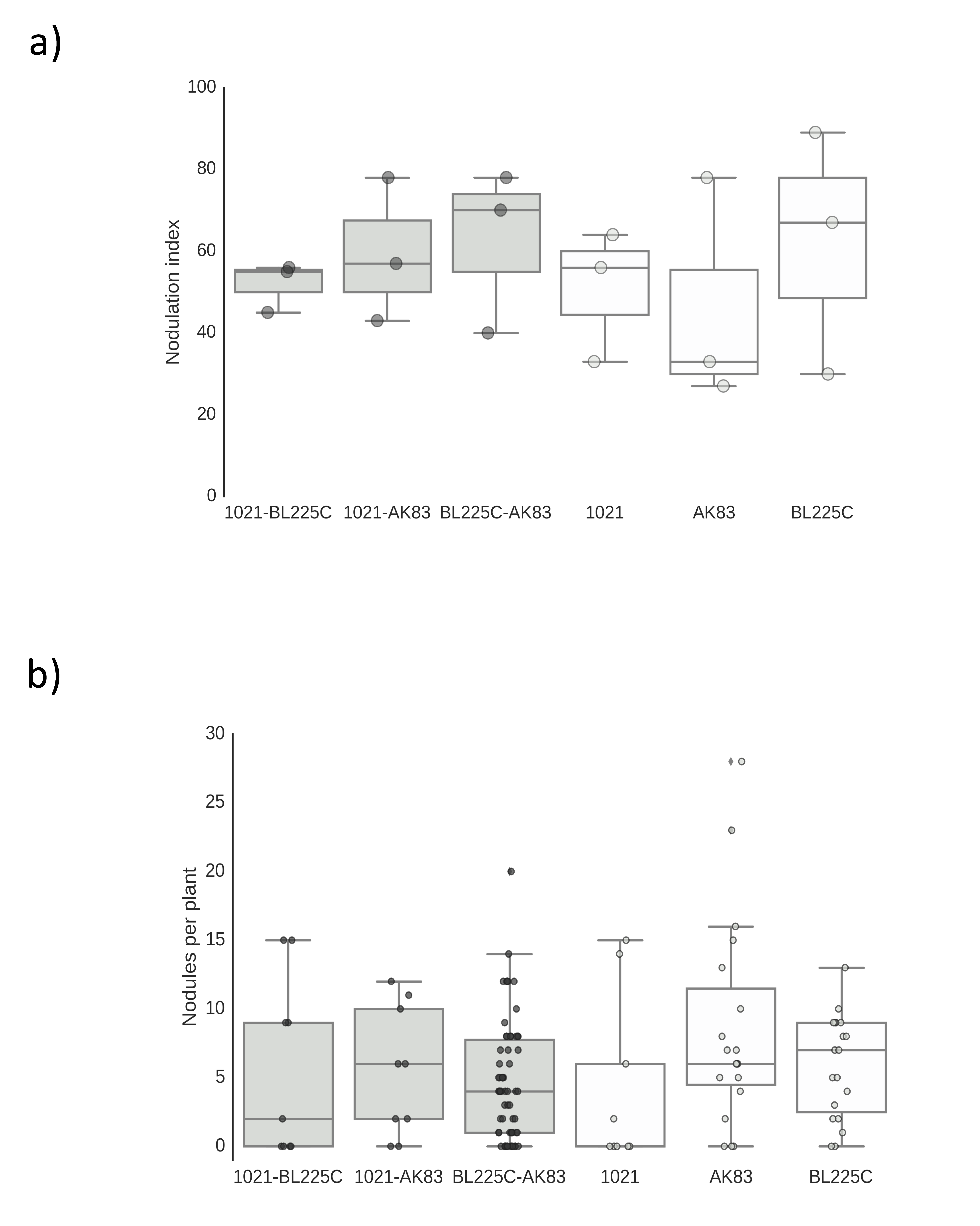

Supplement: FIGURE S2 — Effect of strain competition on host plants. (A) Nodulation index. Percentage of nodulated plants for single and mixed strains combinations. Values are means (śstandard deviation) of three independent experiments, each involving at least 20 plants. (B) Nodulation score. Mean number of nodules/plant. Values indicate means ś standard deviation of number of three independent experiments, each involving at least 20 plants. The only significant pairwise contrast is that AK83 vs. BL225C-AK83 (P < 0.05). [file Image_2.TIF]
