# Supplementary material for: Mixed Nodule Infection in Sinorhizobium meliloti–Medicago sativa Symbiosis Suggest the Presence of Cheating Behavior
Source: Front Plant Sci. 2016 Jun 13;7:835. doi: 10.3389/fpls.2016.00835 (PMC4904023; doi:10.3389/fpls.2016.00835)
Supplement: SUPPLEMENTAL MATERIAL S1 — Input data and code used to generate the figures. [file Data_Sheet_1.ZIP › competition.html]

competition


In [1]:

```
# Plotting imports
%matplotlib inline

import matplotlib.pyplot as plt
import seaborn as sns

sns.set_style('white')

plt.rc('font', size=15)
plt.rc('xtick', labelsize=15)
plt.rc('ytick', labelsize=15)
plt.rc('axes', labelsize=16, titlesize=16)
plt.rc('legend', fontsize=15)
```

In [2]:

```
# Other imports
import pandas as pd
import numpy as np
```

In [3]:

```
f1a = pd.read_table('1a.txt')
```

In [4]:

```
plt.figure(figsize=(10, 6))
ax = sns.boxplot(data=f1a,
                 palette=[sns.xkcd_rgb["grey"],
                        sns.xkcd_rgb["grey"],
                        sns.xkcd_rgb["light grey"],
                        sns.xkcd_rgb["light grey"],
                        sns.xkcd_rgb["grey"],
                        sns.xkcd_rgb["grey"],
                        sns.xkcd_rgb["pale grey"],
                        sns.xkcd_rgb["pale grey"],
                        sns.xkcd_rgb["pale grey"],])
ax = sns.stripplot(data=f1a,
                    size=10, jitter=True, edgecolor="gray",
                    alpha=0.7,
                    palette=[sns.xkcd_rgb['light green'],
                             sns.xkcd_rgb['light blue'],
                             sns.xkcd_rgb['light green'],
                             sns.xkcd_rgb['light red'],
                             sns.xkcd_rgb['light blue'],
                             sns.xkcd_rgb['light red'],
                             sns.xkcd_rgb['light blue'],
                             sns.xkcd_rgb['light red'],
                             sns.xkcd_rgb['light green'],])

plt.axvline(1.5,
            color=sns.xkcd_rgb['grey'],
            linestyle='dashed')
plt.axvline(3.5,
            color=sns.xkcd_rgb['grey'],
            linestyle='dashed')
plt.axvline(5.5,
            color=sns.xkcd_rgb['grey'],
            linestyle='dashed')
plt.xticks(range(9),
           ['BL225C',
            'AK83',
            'BL225C',
            '1021',
            'AK83',
            '1021',
            'AK83',
            '1021',
            'BL225C'])
sns.despine(bottom=True)
plt.yscale('log')
plt.text(.5, 0.01,
         'BL225C/AK83',
         fontdict={'ha':'center'})
plt.text(2.5, 0.01,
         'BL225C/1021',
         fontdict={'ha':'center'})
plt.text(4.5, 0.01,
         'AK83/1021',
         fontdict={'ha':'center'})
plt.ylabel('Viable titres estimates')
plt.savefig('1a.tiff', dpi=300)
```

In [5]:

```
f1b = pd.read_table('1b.txt',
                    header=None)
f1b.columns = ['no', 'no',
               'BL225C',
               'AK83',
               'no', 'no',
               '1021.1',
               'BL225C.1',
               'no', 'no', 'no',
               'AK83.2',
               '1021.2',
               'no', 'no',
               'AK83.3',
               'no',
               '1021.3',
               'no',
               'BL225C.3']
f1b.drop([0, 1], inplace=True)
f1b.drop([x for x in f1b.columns if x == 'no'],
         axis=1,
         inplace=True)
```

In [6]:

```
plt.figure(figsize=(10, 6))
ax = sns.boxplot(data=f1b,
                 palette=[sns.xkcd_rgb["grey"],
                        sns.xkcd_rgb["grey"],
                        sns.xkcd_rgb["light grey"],
                        sns.xkcd_rgb["light grey"],
                        sns.xkcd_rgb["grey"],
                        sns.xkcd_rgb["grey"],
                        sns.xkcd_rgb["pale grey"],
                        sns.xkcd_rgb["pale grey"],
                        sns.xkcd_rgb["pale grey"],])
ax = sns.stripplot(data=f1b,
                    size=10, jitter=True, edgecolor="gray",
                    alpha=0.7,
                    palette=[sns.xkcd_rgb['light green'],
                             sns.xkcd_rgb['light blue'],
                             sns.xkcd_rgb['light green'],
                             sns.xkcd_rgb['light red'],
                             sns.xkcd_rgb['light blue'],
                             sns.xkcd_rgb['light red'],
                             sns.xkcd_rgb['light blue'],
                             sns.xkcd_rgb['light red'],
                             sns.xkcd_rgb['light green'],])

plt.axvline(1.5,
            color=sns.xkcd_rgb['grey'],
            linestyle='dashed')
plt.axvline(3.5,
            color=sns.xkcd_rgb['grey'],
            linestyle='dashed')
plt.axvline(5.5,
            color=sns.xkcd_rgb['grey'],
            linestyle='dashed')
plt.xticks(range(9),
           ['BL225C',
            'AK83',
            'BL225C',
            '1021',
            'AK83',
            '1021',
            'AK83',
            '1021',
            'BL225C'])
sns.despine(bottom=True)
plt.yscale('log')
plt.text(.5, 0.1,
         'BL225C/AK83',
         fontdict={'ha':'center'})
plt.text(2.5, 0.1,
         'BL225C/1021',
         fontdict={'ha':'center'})
plt.text(4.5, 0.1,
         'AK83/1021',
         fontdict={'ha':'center'})
plt.ylabel('qPCR')
plt.savefig('1b.tiff', dpi=300)
```

In [7]:

```
f5 = pd.read_table('5.txt', skiprows=2,
                   header=None).T
f5['strain'] = range(f5.shape[0])
```

In [8]:

```
fig = plt.figure(figsize=(12, 6))
g = sns.barplot(data=-(1 - f5),
                 y=0,
                 x='strain',
                    palette=[sns.xkcd_rgb['light green'],
                             sns.xkcd_rgb['light blue'],
                             sns.xkcd_rgb['light green'],
                             sns.xkcd_rgb['light red'],
                             sns.xkcd_rgb['light blue'],
                             sns.xkcd_rgb['light red'],
                             sns.xkcd_rgb['light blue'],
                             sns.xkcd_rgb['light red'],
                             sns.xkcd_rgb['light green'],])
plt.errorbar(range(f5.shape[0]), -(1 - f5[0]), yerr=f5[2],
             fmt='ko')
plt.axvline(1.5,
            color=sns.xkcd_rgb['grey'],
            linestyle='dashed')
plt.axvline(3.5,
            color=sns.xkcd_rgb['grey'],
            linestyle='dashed')
plt.axvline(5.5,
            color=sns.xkcd_rgb['grey'],
            linestyle='dashed')
plt.xticks(range(9),
           ['BL225C',
            'AK83',
            'BL225C',
            '1021',
            'AK83',
            '1021',
            'AK83',
            '1021',
            'BL225C'])
sns.despine(bottom=True)
ax = fig.axes[0]
ax.set_yticklabels([str(x+1) for x in ax.get_yticks()])
plt.axhline(0,
            color='k')
plt.text(.5, -2.,
         'BL225C/AK83',
         fontdict={'ha':'center'})
plt.text(2.5, -2.,
         'BL225C/1021',
         fontdict={'ha':'center'})
plt.text(4.5, -2.,
         'AK83/1021',
         fontdict={'ha':'center'})
plt.xlabel('')
plt.ylabel('V/T ratio')
plt.savefig('5.tiff', dpi=300)
pass
```

In [9]:

```
f4 = pd.read_table('4.txt',
                   header=None)
f4.columns = ['strain', 'y']
```

In [10]:

```
plt.figure(figsize=(12, 6))
ax = sns.boxplot(data=f4,
                 x='strain',
                 y='y',
                 order=['AK83/BL225C',
                        'BL225C/1021',
                        'AK83/BL225C',
                        'AK83',
                        '1021',
                        'BL225C'],
                palette=[sns.xkcd_rgb['light grey'],
                         sns.xkcd_rgb['light grey'],
                         sns.xkcd_rgb['light grey'],
                         sns.xkcd_rgb['pale grey'],
                         sns.xkcd_rgb['pale grey'],
                         sns.xkcd_rgb['pale grey'],])
sns.stripplot(data=f4,
                 x='strain',
                 y='y',
                 size=10, jitter=True, edgecolor="gray",
                 alpha=0.7,
                 order=['AK83/BL225C',
                        'BL225C/1021',
                        'AK83/BL225C',
                        'AK83',
                        '1021',
                        'BL225C'],
                 palette=[sns.xkcd_rgb['pale grey'],
                         sns.xkcd_rgb['pale grey'],
                         sns.xkcd_rgb['pale grey'],
                         sns.xkcd_rgb['light blue'],
                         sns.xkcd_rgb['light red'],
                         sns.xkcd_rgb['light green'],])

plt.xlabel('')
plt.ylabel('nmoles C2h4/h/plant')
plt.axhline(0,
            color=sns.xkcd_rgb['grey'],
            linestyle='dashed')
sns.despine(bottom=True)
plt.savefig('4.tiff', dpi=300)
```

In [11]:

```
f3a = pd.read_table('3a.txt')
```

In [12]:

```
plt.figure(figsize=(10, 6))
ax = sns.boxplot(data=f3a,
                 color=sns.xkcd_rgb["pale grey"],
                 order=['AK83/1021', 'AK83/BL225C', 'BL225C/1021'])
ax = sns.stripplot(data=f3a,
                    size=10, jitter=True, edgecolor="gray",
                    alpha=0.7,
                    order=['AK83/1021', 'AK83/BL225C', 'BL225C/1021'],
                    palette=[sns.xkcd_rgb['light blue'],
                             sns.xkcd_rgb['light green'],
                             sns.xkcd_rgb['light red']])
plt.hlines(0, -0.5, 3,
          linestyles='dashed',
          alpha=0.5)
plt.ylabel('Viable competition index')
sns.despine(bottom=True)
plt.savefig('3a.tiff', dpi=300)
```

In [13]:

```
f3b = pd.read_table('3b.txt')
```

In [14]:

```
plt.figure(figsize=(10, 6))
ax = sns.boxplot(data=f3b,
                 color=sns.xkcd_rgb["pale grey"],
                    order=['AK83/1021', 'AK83/BL225C', 'BL225C/1021'])
ax = sns.stripplot(data=f3b,
                    size=10, jitter=True, edgecolor="gray",
                    alpha=0.7,
                    order=['AK83/1021', 'AK83/BL225C', 'BL225C/1021'],
                    palette=[sns.xkcd_rgb['light blue'],
                             sns.xkcd_rgb['light green'],
                             sns.xkcd_rgb['light red']])
plt.hlines(0, -0.5, 3,
          linestyles='dashed',
          alpha=0.5)
plt.ylabel('qPCR competition index')
sns.despine(bottom=True)
plt.savefig('3b.tiff', dpi=300)
```

In [15]:

```
s1a = pd.read_table('s1a.txt', header=None)
s1a = s1a.T
s1a.columns = s1a.iloc[0]
s1a = s1a.reindex(s1a.index.drop(0))
s1a = s1a.drop(s1a.columns[0], axis=1)
```

In [16]:

```
plt.figure(figsize=(10, 6))
ax = sns.boxplot(data=s1a,
                 palette=[sns.xkcd_rgb["light grey"],
                        sns.xkcd_rgb["light grey"],
                        sns.xkcd_rgb["light grey"],
                        sns.xkcd_rgb["pale grey"],
                        sns.xkcd_rgb["pale grey"],
                        sns.xkcd_rgb["pale grey"]])
sns.stripplot(data=s1a,
              size=10, jitter=True, edgecolor="gray",
              alpha=0.5,
              palette=[sns.xkcd_rgb["dark grey"],
                        sns.xkcd_rgb["dark grey"],
                        sns.xkcd_rgb["dark grey"],
                        sns.xkcd_rgb["light grey"],
                        sns.xkcd_rgb["light grey"],
                        sns.xkcd_rgb["light grey"]])
plt.xlabel('')
plt.ylabel('Nodulation index')
plt.ylim(0., 100)
sns.despine(bottom=True)
plt.savefig('s1a.tiff', dpi=300)
```

In [17]:

```
s1b = pd.read_table('s1b.txt')
```

In [18]:

```
plt.figure(figsize=(10, 6))
ax = sns.boxplot(data=s1b,
                 palette=[sns.xkcd_rgb["light grey"],
                        sns.xkcd_rgb["light grey"],
                        sns.xkcd_rgb["light grey"],
                        sns.xkcd_rgb["pale grey"],
                        sns.xkcd_rgb["pale grey"],
                        sns.xkcd_rgb["pale grey"]])
sns.stripplot(data=s1b,
              size=5, jitter=True, edgecolor="gray",
              alpha=0.7,
              palette=[sns.xkcd_rgb["dark grey"],
                        sns.xkcd_rgb["dark grey"],
                        sns.xkcd_rgb["dark grey"],
                        sns.xkcd_rgb["light grey"],
                        sns.xkcd_rgb["light grey"],
                        sns.xkcd_rgb["light grey"]])
plt.xlabel('')
plt.ylabel('Nodules per plant')
sns.despine(bottom=True)
plt.ylim(-1., 30.)
plt.savefig('s1b.tiff', dpi=300)
```
